# Evaluation of the effectiveness of “Shega” natural and self-made solution as compared to permethrin lotion in eliminating head lice in infested Schoolchildren in Gondar area, Ethiopia: a randomized non-inferiority trial

**DOI:** 10.3389/fped.2025.1507760

**Published:** 2025-03-28

**Authors:** Tewodros Ayalew Tessema, Anne-Laure Cavin, Abyot Endale Gurmu, Liknaw Workie Limenh, Gizachew Kassahun Bizuneh, Betelhem Anteneh Adamu, Annisa Befekadu, Henok Dagne, Bertrand Graz

**Affiliations:** ^1^ Department of Pharmaceutics, School of Pharmacy, College of Medicine and Health Sciences, University of Gondar, Gondar, Ethiopia; ^2^ Antenna Foundation, Geneva, Switzerland; ^3^ School of Pharmaceutical Sciences, University of Geneva, CMU, Geneva, Switzerland; ^4^ Department of Pharmacognosy, School of Pharmacy, College of Medicine and Health Sciences, University of Gondar, Gondar, Ethiopia; ^5^ Traditional and Modern Medicine Research and Development Directorate, Armauer Hansen Research Institute, Addis Ababa, Ethiopia; ^6^ Department of Dermatology and Venereology, College of Medicine and Health Sciences, University of Gondar, Gondar, Ethiopia; ^7^ Department of Environmental and Occupational Health and Safety, College of Medicine and Health Sciences, University of Gondar, Gondar, Ethiopia

**Keywords:** pediculosis, school children, head lice infestation, Shega, natural solution

## Abstract

**Background:**

Head lice infestation among schoolchildren leads to social stigmatization, psychological distress, superinfection, and lack of concentration because of sleeplessness. Head lice infestation is universal and not found solely in privileged populations. Although there are modern medicines for the treatment of head lice, most communities are unable to access them due to high prices, limited supply in remote areas, or lack of willingness to use them because of the negative attitudes towards head lice infestation amongst children and the community. Therefore, this study assessed the effectiveness of a homemade remedy (“Shega solution”) made with easily available ingredients at a low cost as compared to the standard Permethrin 1% lotion.

**Method:**

The study was conducted in the Amhara region of Ethiopia in five schools in Gondar city and the surrounding district. The selected school children were randomly assigned to intervention (Shega) and control (Permethrin) groups. The study was designed as a non-inferiority trial, with the hypothesis that Shega would, at worst, be only marginally inferior to standard treatment, by a margin (=delta) not exceeding 30 percentage points. A theatre play was created and performed in front of the whole school community to familiarize the school children with the study and to remove stigmatization about head lice infestation.

**Results:**

Three hundred and eighty-four schoolchildren were included in the study. About 67.7% of participants were cured in the intervention group compared to 83.3% in the control group. The difference of 15.6 points of percentage is within the set acceptable range, with a 95% CI difference in the proportion of success of 7.2–24.1 for the difference in success rates. The theatrical event was noted as useful in breaking the social stigma and familiarizing the children with the research project.

**Conclusion:**

The study has shown that the homemade remedy, Shega solution, has the potential to treat a fair proportion of head lice infestations as compared to the standard treatment, permethrin. The theatre performed in front of the school children has the potential to help the implementation of such projects at community levels. This study also indicates the level of effectiveness of permethrin lotion in Ethiopia for the first time.

**Clinical Trial Registration:**

https://pactr.samrc.ac.za/Search.aspx, identifier (PACTR202208887378021).

## 1 Introduction

Head lice (*Pediculus humanus var. capitis*) are wingless insects that are only found in humans, and nourish themselves solely with blood. The head louse hatches long, pale gray to reddish brown oval-shaped eggs (nits), 0.3–0.8 mm in size. The female lice attach the eggs to the hair, about 1–3 cm from the scalp, at the neck, or behind the ears by preference, using strong water-proof cement (1). The saliva of head lice induces skin irritation and might lead to superinfection from scratching, with related social stigmatization, psychological distress, and lack of concentration in children because of sleeplessness. Moreover, head louse infestation may cause high levels of anxiety among the parents of school-aged children.

A cross-sectional study using semi-structured questionnaires conducted by Dagne et al. (2) revealed that the prevalence of head lice among schoolchildren in Woreta town, Ethiopia was 65.7%, and concluded that head lice infestation is a major public health problem and needs intervention.

Different medicines were developed to alleviate problems related to head lice infestation. The first generation of anti-lice treatments, developed in the forties, had a neurotoxic action. Those treatments are essentially composed of organophosphate insecticides (malathion) and pyrethroids (e.g., pyrethrins, phenothrin, and permethrin). The American Academy of Pediatrics recommends Permethrin 1% lotion, which is still the first-line pharmacologic treatment of pediculosis in some areas of the world due to its safety and efficacy (3). Although Permethrin lotion is the first-line treatment for head lice in Ethiopia, no data regarding its efficacy in Ethiopia have been reported so far. Moreover, it is not affordable for a large proportion of the community and is most often not available in rural areas. Most probably, resistance to this pediculicide will occur in the next decades, as it happened elsewhere (4).

This highlights the need to develop a new, safe, efficient, and affordable alternative. Indeed, an inexpensive homemade solution has been developed by a research group (5); it was made of table salt, vinegar, and a wetting agent in water.

The success rate of this solution was first evaluated in a pilot study in a school in Brazil, where the solution was associated with 55.2% success, with no use of an anti-lice comb (5). A study with three versions of this solution has been conducted by the same research group in Nepal with satisfactory results: 71.1% of participants got rid of head lice with the homemade solutions, 14.4% more lice elimination than the standard Nepalese treatment mustard oil (6).

In the present study, one of these solutions, called here Shega was assessed on frizzy/curly hair without the use of a lice comb (as its use is too difficult with such hair texture) and in comparison, with the standard treatment Permethrin.

In addition, as a secondary outcome the effectiveness of Permethrin 1% lotion, was applied three times without the use of an anti-lice comb and to evaluate the possible side effects of both solutions.

## 2 Methods and materials

### 2.1 Methods

#### 2.1.1 The study site and population

The study was conducted in five schools in Gondar City and the surrounding district of the Amhara region of Ethiopia from **15/10/2022 to 25/6/2023**. Four schools were included from Gondar City, where the local partner, the University of Gondar is situated, and one school, Maksegnit Town, 40 km south of Gondar City.

The recruitment of the study population which included identifying eligible study participants, conducting discussions with parents, and consent forms from parents were obtained from **15/10/2022 to 15/2/2023**. The actual study was conducted from **25/2/2023 to 25/6/2023**.

A preliminary prevalence survey on head lice infestation was conducted in the five selected schools before the performance of the theatrical events and discussions with parents, which was the first step of this study.

Once discussion sessions with the parents were completed, they were asked to sign the written consent form.

### 2.2 Materials

#### 2.2.1 Shega solution

The active ingredients of the Shega solution are table salt (5 g), vinegar (1 ml), and glycerin in water (Table 1). Table salt (purity 99.9%), plastic bags, cloth, and vinegar (5% acetic acid) were bought from the local Ok Asbeza supermarket in Gondar. The quality of the table salt was further confirmed in the pharmaceutical chemistry laboratory of the University of Gondar. Plastic containers were bought from Ya Water Factory plc, Gondar, while soap, vinegar, Glycerin (purity 99%), normal combs, cotton rolls, and alcohol were bought from Arsema Pharmacy, Gondar. Anti-lice combs were bought from an online store, *Yangzhou Jianxin Brush-Making Company Ltd., China*. Permethrin lotion (1%) was purchased from the local Admas pharmaceutical wholesaler, Bahir-Dar. The labeling was designed and printed in Zelalem Printing House in Gondar. The measuring cylinder, stirrer, and funnels were borrowed from the University of Gondar.

**Table 1:** Composition of Shega solution.

Ingredients	Amount
Salt	5 g
Vinegar	1 ml
Glycerin	5 ml
Water	To 100 ml

#### 2.2.2 Study design

The study was designed as a non-inferiority randomized controlled trial with a ratio of 1:1 in each group, with a delta of 0.3 (pre-defined limit of non-inferiority, maximum difference considered acceptable from a public health point of view).

#### 2.2.3 Inclusion criteria

School children from grades 1 to 8 and between 6 and 14 years old having head lice infestation (both egg and adult) (detected by visual inspection with the help of a normal comb) and whose parents signed the written consent forms were included in the study.

#### 2.2.4 Exclusion criteria

Children with scalp wounding or any chronic disease were excluded. Children whose parents didn't give written informed consent were also excluded from the study.

### 2.3 Sample size

Expecting a success rate of 75% for the Permethrin group and 55% for the Shega group, thus a difference of 20 points of percentage, and defining “non-inferiority” as a difference between the success rates of less than 30 points of percentage, the estimated sample size, based on the value of the upper limit of the 95% confidence interval (one-sided α error of 2.5%), was 174. With subsequent corrections to account for potential attrition (10%), the total required sample at inclusion was 384 schoolchildren (192 in each study group).

### 2.4 Randomization

The randomization process was conducted using the flipping coin method, where the heads and tails of the coin were assigned as control and treatment groups, respectively (7). While flipping a coin, the number required for the head (control group) was satisfied (192) before the completion of the process, so the rest of the students were assigned to the treatment group (192). Once the allocation of the school children to both groups was completed, every 15 students were registered to one of the working teams delegated to assist in the process. The randomization process was conducted independently by the research supervisors who were left blinded. However, due to the nature of the application process, the care providers and patients were not kept blind.

### 2.5 Study variables

The study mainly focuses on the evaluation of the effectiveness of the Shega Solution as compared to the Permethrin Solution. The outcome variables were “cure” or “not cure” after evaluating using an anti-lice comb. If any number of lice or eggs were detected during the evaluation phase, then the result was labeled as not cured. If nothing was detected, then it was noted as cured.

### 2.6 Quality analysis of table salt

As sodium chloride is produced locally, to avoid any quality-related issues in the formulation of Shega solution for the study, the ingredients were assessed for their quality in the pharmaceutical analysis laboratory of the University of Gondar. The assay was conducted by an analyst as per the method presented in the British Pharmacopeia (BP, 2007). 0.2 g of table salt (NaCl) was weighed accurately, dissolved in water up to 50 ml, and then titrated with 0.1 M silver nitrate. The endpoint was determined potentiometrically. Each ml of 0.1N silver nitrate is equivalent to 5.844 mg of sodium chloride (NaCl).

### 2.7 Fieldwork organization

To establish a climate of trust, the fieldwork started with the organization of an inception workshop dedicated to all concerned stakeholders, including the school directors, members of the zonal and district health departments, the University of Gondar (UoG), and zonal and district education departments. Then, once the study was well understood and all clarifications made, a second workshop for parents (caregivers) and the school community, including community leaders and area administrators, was organized in each school to explain in detail the steps of the trial and to obtain their full commitment. The next step was to select a working team among teachers from each school, with a ratio of 1 teacher for 15 children. They were trained about each step of the project implementation. Parallel to this, a theatre group was organized amongst students from the Theatrical Department of the University of Gondar. The theatrical team wrote the theatrical presentation about head lice which were performed for children to remove stigma and open dialogue. Once all workshops had been completed, the theatre group performed in front of the whole community (schoolchildren, teachers, parents).

### 2.8 Intervention

As part of the initial process, screening for head lice (adults and eggs) was conducted among schoolchildren using a normal comb. All of the school teachers were trained about how to screen the head lice to make sure that lice are sufficiently investigated as they can hide at the skin of the skull while not visible superficially. The training was delivered by the project supervisors from the UoG using leaflets and other reading materials about head lice, their life cycle, and the methods of detection. The training also included a brief introduction to data recording and management to ensure high-quality data.

Then, parents of the infested children were called for an explanation about the study. Those children whose parents agreed and signed the consent form were included in the project and then randomly assigned to the standard/control (Permethrin) or treatment (Shega solution) group from February to June 2023. Both medicines were applied by trained working staff at each school under the supervision of project facilitators from the UoG.

The treatment protocol for the Shega solution was as follows: the hair was untangled, and the Shega solution was applied to dry hair until it became wet. After an hour, it was combed with a normal comb and left for 24 h without washing. This step was conducted three times every 7 days (Day 0, Day 7, and Day 14).

For Permethrin lotion: The hair was untangled, and the Permethrin lotion was applied on damp hair, then left for 10 min. After this, the hair was combed with a normal comb and rinsed thoroughly after 10 min of application. This was done 3 times every 7days (Day 0, Day 7 and Day 14).

For children of both groups, recommendations about secondary measures that should be done at home were made: treatment of infested sibling with the standard treatment Permethrin, frequent delousing of clothes with hot water and soap, and for what could not be washed, placed in a plastic bag for 2 days.

### 2.9 Data collection

The 16th day was a control day wherein each child's head was examined both visually and with a lice comb and white cloth for the presence of head lice. This was done by a trained working staff from each school under the supervision of facilitators from the University of Gondar. Then, supervisors check on the white cloth to see whether the head lice and/or eggs fall on it. In addition, the data questionnaires were filled out by trained staff from the University of Gondar immediately after the examination on the control day, and students were interviewed for their satisfaction and the presence of some adverse effects in each of the applications as per the questionnaire.

### 2.10 Statistical analysis

Data were double-entered in Excel tables, and analysed with Epi Info7 and STAT (version 14) in parallel by UoG and Antenna teams.

Demographic data (age, sex, region), and infestation status (prevalence), were summarized for all included school children.

Data on treatment and side effects were summarized globally and by treatment group. Data were analyzed using the intention to treat principle; the chi2 test was to be used to check that the rates of attrition were approximately the same in both treatment groups and thus that missing data would be considered as “at random” but it happened that no one was lost during follow-up and no one changed group.

The success rates (absence of head lice) were estimated and the difference between success rates was calculated. The 95% confidence interval for the difference between the two success rates was estimated using Wald approximation. If the upper limit of the 95% confidence interval (B) were lower than 30 points of percentage (the limit for considering Shega as “non-inferior” to Permethrin), we would conclude the non-inferiority of the Shega treatment. Otherwise, it would be concluded that the success rate with Shega could be inferior to that of Permethrin if there is a difference of more than 30 points of percentage between the two success rates.

### 2.11 Human subject approval statement and trial registry

An ethical clearance was issued by both the Federal Ministry of Education and the University of Gondar with reference numbers **03/246/97/22** and **VP/RTT/05/11/4/2022**, respectively. In addition, support letters were obtained from the Amhara Region Public Health Institute, Central Gondar Zone Health Department, Gondar City Health Administration, Central Gondar Zone Education Office, and Gondar City Education Office. To clarify the study to the caregivers, we have conducted discussions with question-and-answer sessions In addition, we provided written materials with the trial's objectives, procedures, possible risks, and actions that would be taken. The caregivers were also allowed to sign the consent forms before the study, indicating that a child could withdraw from the study at any time and without any penalty. The study was conducted following the revised declaration of Helsinki (2013) and was registered to the Pan-African Clinical Trial Register (No PACTR202208887378021) available at: https://pactr.samrc.ac.za/Search.aspx.

## 3 Results

A total of 5,833 students from five schools (6–15 years old) were screened in the program. It was found that 768 students (13.2%) had head lice. The prevalence of infestation was distributed as follows: Atikilt school: 18.6% (98/526 students), Kilil Rufael school: 17.0% (112/659), Teda school: 13.1% (144/1,098), Makisegnit school 9.6% (241/2,520) and Yemariam Debir school: 16.8% (173/1,030). The mean prevalence of head lice among girls was 21.2% (from 14.3% to 31.5% according to the school) and 4.9% among boys (from 3.6 to 6.9%).

Out of the 768 affected schoolchildren, 384 were **randomly** enrolled in the present study as requested by the sample size calculation. At the final Control Day (D16/17), none was lost to follow-up, thus 384 were enrolled in the final analysis, and amongst them 85.4% (328/384) were girls. The age of all school children fell between 6 and 15 years, with a mean of 10.5. The 2 treatment groups were similar in terms of gender (82.8 vs. 88% girls – *p* = 0.14 and age (10.8 vs. 10.3-year-old) in permethrin and shega groups.

### 3.1 The success rates of the two solutions

**Figure 1:**
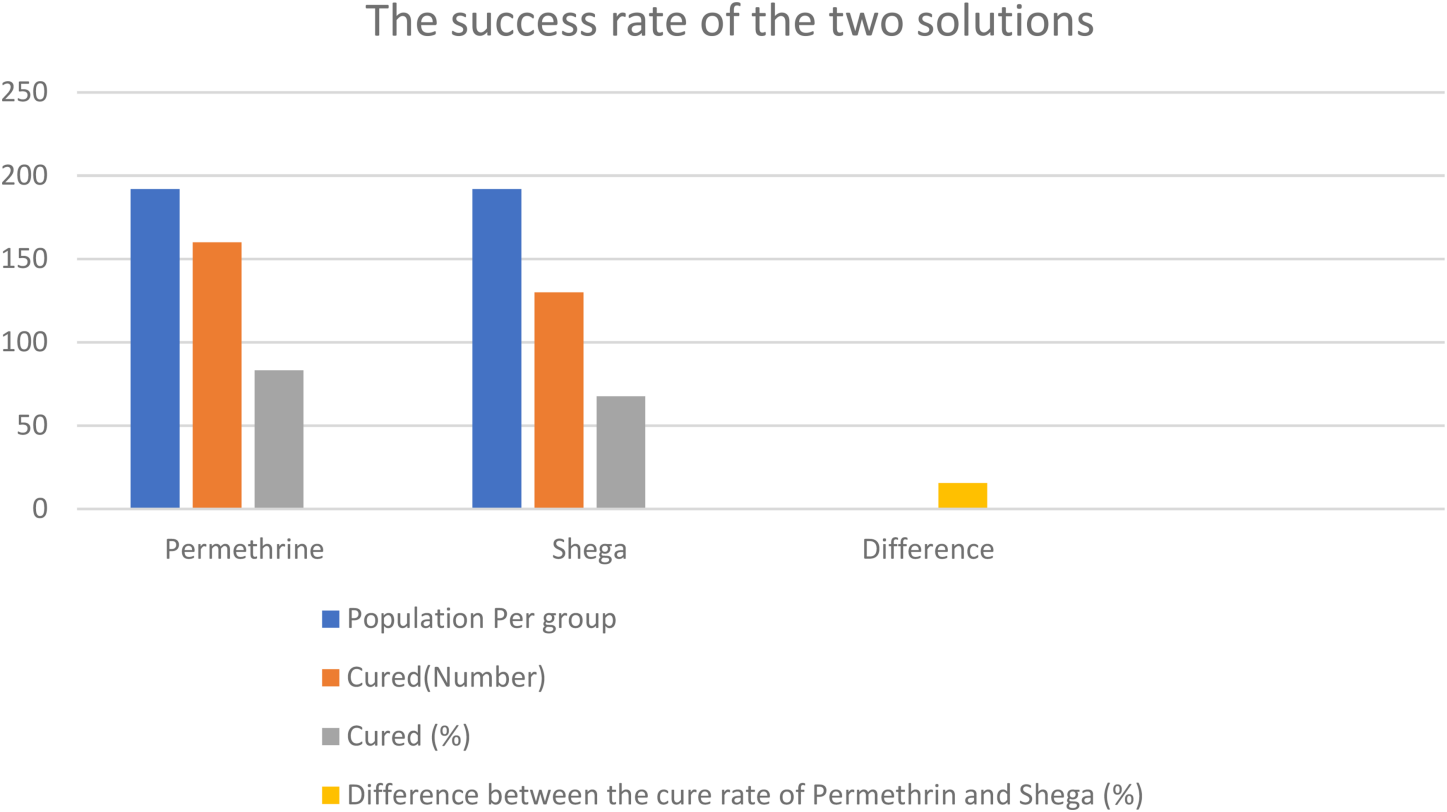
The success rate of the two groups.

Of the study participants who took the standard (permethrin) 83.3% were cured while **67.7%** of those who took Shega Solution were cured. The difference between the success rates was 15.6 points percentage, the lower limit of the 95% CI was 7.2 and the upper limit was 24.1. The non-inferiority condition was fulfilled (Figure 1).

### 3.2 Side effects related to the products

In assessing any adverse effects during and after applications, among 192 students who took permethrin, four reported a persistent burning sensation that lasted for about 1 h after the application of permethrin. In contrast, 10 among 192 students who took Shega Solution had an instant burning sensation that appeared at the time of the application and disappeared after 5–10 min of the completion of the application. In addition, four students in the Shega group reported they had a mild headache while they took the solution.

### 3.3 Quality analysis of table salt

The table salt was found to have 102% (±5%) sodium chloride content which passed the test as confirmed compared with the reference which is 95%–105% (8).

## 4 Discussion

The World Health Organization urged continuous research and development for the treatment of head and body lice due to the emerging resistance to pyrethroid and organophosphate insecticides such as permethrin and Malathion (4, 9, 10).

So far, efforts have been made to find natural solutions for head lice. For example, grapefruit extract was proven effective in a study conducted in Egypt (11). In another clinical trial study, a natural head lice solution called MOOV was compared with pyrethrin, and its efficacy was reported to be better than that of organophosphate compounds (12). *in vitro* studies on essential oils such as tea tree oil have also been reported to have an appealing result in headlice treatment (13).

In this study, Shega solution appears non-inferior to permethrin lotion. The non-inferiority criterion for the difference in rates of success between the permethrin and Shega solution was set at 30 points of percentage. The observed difference between rates of success was 15.6. The lower limit of the 95% CI is superior to zero (7.2). Thus, it must be concluded that the difference is statistically significant. The upper limit of the 95% CI is 24.1, which is less than the fixed non-inferiority criteria. Since the difference in success rates between the Permethrin and Shega solution is inferior to 30, the Shega solution appears to have an acceptable effectiveness compared to Permethrin. As Shega presents several advantages – it is made of natural and cheap ingredients that are available in almost every setting – its slightly lower efficiency is therefore balanced by its accessibility, durability, and low cost.

The burning sensation after the application of permethrin is probably due to the product, as burning sensations have been reported (14). On a similar note, the burning sensation related to the Shega solution might be due to the presence of salt or vinegar in the solution and its adherence to the scalp because of the presence of glycerin (15).

The mild headache reported by 4 students in the Shega group might be related to fear of taking the solution as there were rumors that the new solution could affect the mental functioning of students, which was clarified with discussions with the parents and school community before the start of the project.

## 5 General satisfaction

The theatre performed in front of the students was found interesting and instrumental in attracting students to the study by avoiding the stigma. All of the schoolchildren said “yes” when asked whether they were happy with the theatrical event. Moreover, the students and their families appreciated the inclusion of the theatre and discussions in the program, which gave them a space to understand the importance of the research. Furthermore, they mentioned instructions they received to prepare the remedy at their home made them happy and gave them hope in increasing their quality of life.

## 6 Limitation of the study

The schoolchildren and parents were unable to strictly adhere to the protocol measures such as regular washing at home. This is mainly due to a lack of water to wash their hair and clothes and ignorance of the instructions, such as the use of plastic bags for keeping infected cloth for 48 h to get rid of lice, which might have contributed to the cure rates of the products employed (Shega or Permethrin) in this study.

## 7 Conclusion

This study shows the efficacy of Shega solution, as non-inferior to the standard treatment, for the treatment of head lice infestation among school children.

About the side effects observed: some of the students reported burning sensations, which is expected due to the salty or vinegar content of the Shega solution. These reactions were resolved rapidly as the solution dried.

Therefore, the homemade Shega solution appears to have an acceptable effectiveness for treating head lice infestation and can be used by the communities to prepare and treat themselves when required.

## Data Availability

The raw data supporting the conclusions of this article will be made available by the authors, without undue reservation.
