# Supplementary material for: Evaluation of the effectiveness of “Shega” natural and self-made solution as compared to permethrin lotion in eliminating head lice in infested Schoolchildren in Gondar area, Ethiopia: a randomized non-inferiority trial
Source: Front Pediatr. 2025 Mar 28;13:1507760. doi: 10.3389/fped.2025.1507760 (PMC11998915; doi:10.3389/fped.2025.1507760)
Supplement: Supplementary file 1 [file Image1.pdf]

CONSORT

TRANSPARENT REPORTING of TRIALS

Title “Evaluation of the Effectiveness of "Shega" Natural and Self-made Solution as Compared to Permethrin Lotion in Eliminating Head lice in Infested School-Children in Gondar area, Ethiopia: A Randomized non-inferiority Trial.

CONSORT 2010 Flow Diagram

Enrollment

Assessed for eligibility (n=5833)

Excluded (n= 5449)
- Not meeting inclusion criteria (n= 5336)
- Declined to participate (n=113)
- Other reasons (n=0)

Randomized (n= 384)

Allocation

Allocated to Permethrin group (n= 192)
- Received allocated intervention (n= 173)
- Did not receive the allocated intervention (give reasons)(n= 19)

Among the three applications planned to be conducted on the 0, 7th, and 14th day, 19 participants did not take the last dose (14th day). The reason they provided was the second dose cured them and did not need any more treatment. Consequently, the research supervisors accepted their reasons by checking their heads with an anti-lice comb as per protocol and ensuring that they were clean of head lice including eggs. However, these participants were rechecked on the evaluation day (16th) as per the final assessment schedule and their results were included for analysis.

Allocated to Shega group (n= 192)
- Received allocated intervention (n= 185)
- Did not receive the allocated intervention (give reasons) (n= 7)

Among the three applications planned to be conducted at the 0, 7th and 14th day, 7 participants did not take the middle dose (7th day). The reasons for this are not related to the study as the four students (participants) mentioned that they were helping their parents and the rest mentioned they were late to come to school that day.

Enrollment process and Follow-Up

Enrolment process

Once the theater was performed to familiarize students with the study, parents signed the consent form. Then, the flipping coin method was used to assign heads(control) and tails (treatment) groups until our sample size was satisfied.

Follow up process

A trained supervisor's team was established to ensure the study was going as per the protocol. These teams are school-based (established in each school) and comprise members from teachers, community leaders, parent representatives, and experts from the University of Gondar. Their role includes ensuring children adhere to the protocol.

Lost to follow-up (give reasons) (n= 0)

Discontinued intervention (give reasons) (n= 0)

Enrolment process

Once the theater was performed to familiarize students with the study, parents signed the consent form. Then, the flipping coin method was used to assign heads(control) and tails (treatment) groups until our sample size was satisfied.

Follow-up process

A trained supervisor's team was established to ensure the study was going as per the protocol. These teams are school-based (established in each school) and comprise members from teachers, community leaders, parent representatives, and experts from the University of Gondar. Their role includes ensuring children adhere to the protocol

Lost to follow-up (give reasons) (n= 0)

Analysis

Analysed (n= 192)

♦ **Excluded** from analysis (give reasons) (n=0) As the research was conducted at school children within a short period (a total of not more than 20 days starting from application to data collection as in the protocol), children and their parents and teachers were highly engaged in the participation of the research which they reported it as entertaining). Therefore, complete adherence was achieved.

Analysed (n= 192)

♦ **Excluded** from analysis (give reasons) (n=0)

As the research was conducted at school children within short period (total for not more than 20 days starting from application to data collection as in the protocol), children and their parents and teachers were highly engaged in the participation of the research which they reported it as entertaining. Therefore, complete adherence was achieved.
